# Complex correlations between microstructure and magnetic behavior in SrFe_12_O_19_ hexaferrite nanoparticles

**DOI:** 10.1038/s41598-021-02782-2

**Published:** 2021-12-02

**Authors:** Pierfrancesco Maltoni, Sergey A. Ivanov, Gianni Barucca, Gaspare Varvaro, Davide Peddis, Roland Mathieu

**Affiliations:** 1 Department of Materials Science and Engineering, Uppsala University, Box 35, 751 03 Uppsala, Sweden; 2 Department of Chemistry, M.V. Lomonosov Moscow State University, Leninskie Gory 1/3, Moscow, Russia 119991; 3 Department SIMAU, Università Politecnica delle Marche, Via Brecce Bianche 12, 60131 Ancona, Italy; 4 Istituto di Struttura della Materia-CNR, nM2-Lab, 00015 Monterotondo Scalo, RM Italy; 5 Department of Chemistry and Industrial Chemistry, nM2-Lab, Università degli Studi di Genova, Via Dodecaneso 31, 1-16146 Genova, Italy

**Keywords:** Materials science, Nanoscience and technology

## Abstract

The magnetic properties of SrFe_12_O_19_ (SFO) hard hexaferrites are governed by the complex relation to its microstructure, determining their relevance for permanent magnets´ applications. A set of SFO nanoparticles obtained by sol–gel self-combustion synthesis was selected for an in-depth structural X-Rays powder diffraction (XRPD) characterization by means of *G(L)* line-profile analysis. The obtained crystallites´ size distribution reveal a clear dependence of the size along the [001] direction on the synthesis approach, resulting in the formation of platelet-like crystallites. In addition, the size of the SFO nanoparticles was determined by transmission electron microscopy (TEM) analysis and the average number of crystallites within a particle was estimated. These results have been evaluated to illustrate the formation of single-domain state below a critical value, and the activation volume was derived from time dependent magnetization measurements, aiming to clarify the reversal magnetization process of hard magnetic materials.

## Introduction

**Figure 1:**
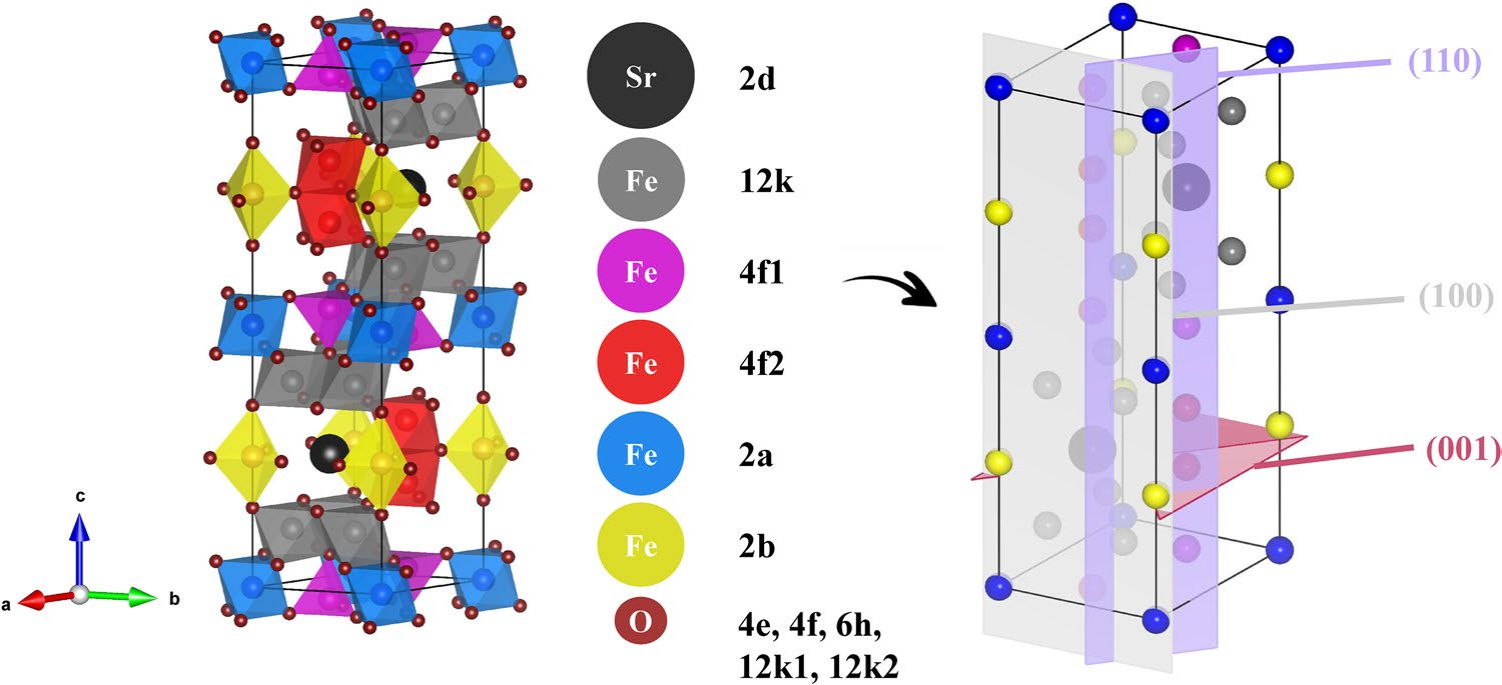
Illustration by VESTA^13^ of the unit cell of SrFe_12_O_19_ (space group *P6*_3_*/mmc*).

Magnetic materials at the nanoscale are of huge scientific and technological interest, as their magnetic properties display remarkably different behavior compared to bulk-size, thus leading to new perspectives and applications^1–4^. Among nanostructured materials, M-type hexaferrite SrFe_12_O_19_ (SFO) has become extremely appealing as promising candidate material for the renewal of permanent magnets applications^5^. As a matter of fact, over the recent years, an intense research effort has been carried out to tailor SFO-based materials at the nanoscale through several synthetic and processing approaches to optimize size, morphology, as well as magnetic properties^6–8^. Moreover, it has received significant attention for the study and development of exchange coupled systems^9,10^. Its high magnetocrystalline anisotropy (*K* = 0.35 MJ/m^3^) directed along the c-axis^11,12^of its hexagonal lattice, is the direct outcome of the complex correlation between magnetic and crystalline structure, crystallite and particle size, morphology and texture. Consequently, controlling the as mentioned features is fundamental to meet specific requirements. Figure 1 illustrates the hexagonal space group *P6*_*3*_*/mmc* typical of SFO^13^, together with the planes corresponding to the reflections investigated by line-profile analysis.

**Figure 2:**
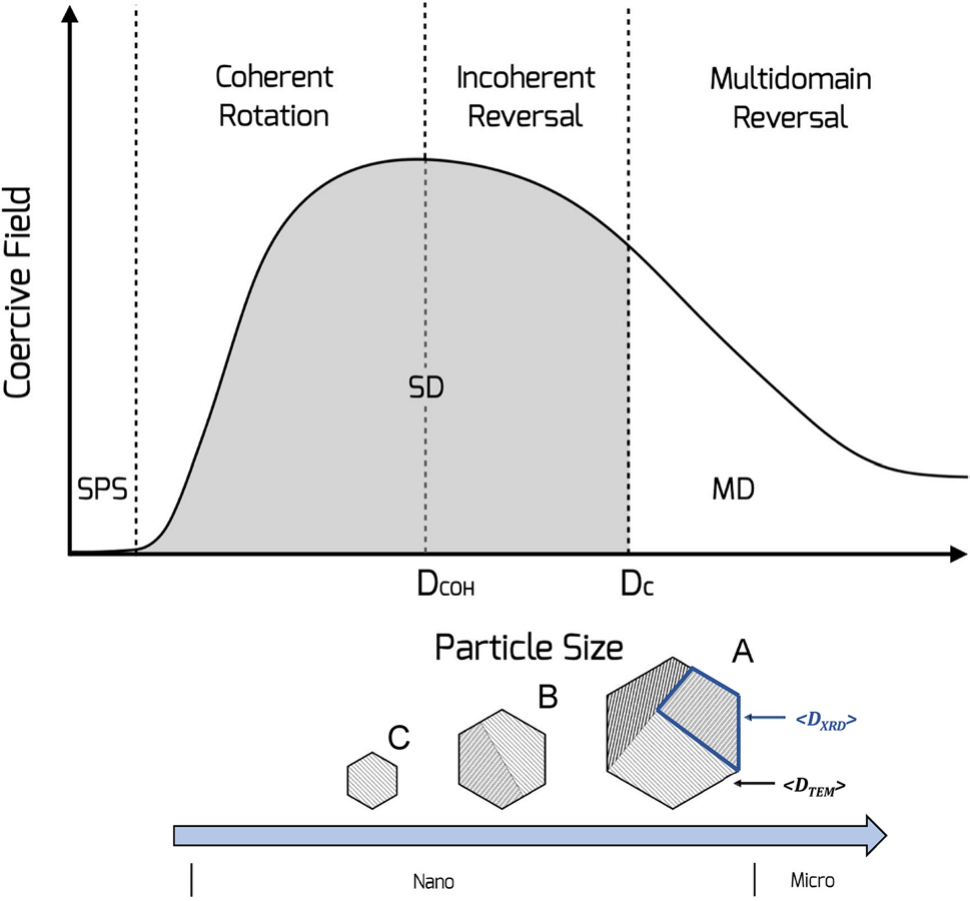
Top: Schematic illustration of the evolution of coercive field as a function of particle size showing the corresponding magnetization reversal processes (adapted from^15^). SPS, SD, and MD stand for superparamagnetic state, single-, and multi-domain, respectively; *D*_*COH*_ and *D*_*C*_ for coherent and critical diameter, respectively. Bottom: Particles sketch with different size showing the growth of crystallites from single to polycrystalline.*<D*_*XRD*_> and *<D*_*TEM*_*>* indicate the crystallite and particle size, respectively.

Among the relevant peculiarities stemming from a size reduction of ferromagnetic particles, the formation of single-domain state below a critical value results in an increase of magnetic anisotropy (owed to the higher surface area to volume ratio) and thus the coercive field^14,15^. A broad region can be identified below the critical size (*D*_*C*_) in a hard material (with typical values around 1 µm), and delimited by the so called coherent size (*D*_*COH*_)^16^: this refers to the minimum volume that demagnetize in a coherent way, denoted as activation volume (*V*_*ACT*_)^14^. However, as displayed in Fig. 2, although the crystal size is smaller than *D*_*C*_, the reversal process might be incoherent. In a nanoparticle (NP) assembly the critical volume for reversal is dependent upon the magnetic viscosity (*S*), whose magnetic field dependence provide important information about the switching process of NPs magnetization^17,18^.

Nevertheless, in the nanoscale, new complex aspects are also introduced, such as strong inter- particle magnetic interactions, size distribution, particles’ shape, surface disorder, orientation of magnetization easy axes, which all make the analysis more challenging^19,20^. These elements significantly affect the energy barrier distribution, and deserve careful consideration, as consequently influence the magnetization reversal mode. On this basis, a correct understanding of the correlation between magnetic volume and physically nanostructured M-type hexaferrite SrFe_12_O_19_ is rather important. Therefore, as a model system, we use a set of SFOs prepared by bottom up sol–gel approach and recently investigated^6^. Previous results indicate that the crystallites’ size lies in the nanoscale range and that it depends, together with the crystallites’ shape, on the employed thermal treatment. In addition, the crystallinity of such samples depends on the synthesis method, and more detailed analysis is needed, to clarify the relation between crystallite and particle size. To disclose this relationship, a careful analysis of crystalline microstructure parameters (i.e., crystallite and particle size, shape) was performed by transmission electron microscopy (TEM) analysis and by combining Rietveld method and line profile analysis of high statistics X-Ray powder diffraction (XRPD) patterns. The structural characterization aims at ascertaining the anisotropic feature of the obtained nanocrystallites, as well as evidencing the feasibility of line-profile analysis as robust technique to characterize peak broadening into the nanoscale range of (ferrite) materials. The volume-weighted crystallite size distribution *G(L)* was found to strongly depend on crystallographic direction. In this work we show that complementary techniques are indeed needed to extract accurately size-dependent parameters to precisely describe the structural and magnetic features of this type of powder samples. The reversal magnetization process is also investigated, to clarify the relation between morpho-structural characteristics and magnetic behavior.

## Results and discussion

### Particle versus crystallite size and morphology

**Figure 3:**
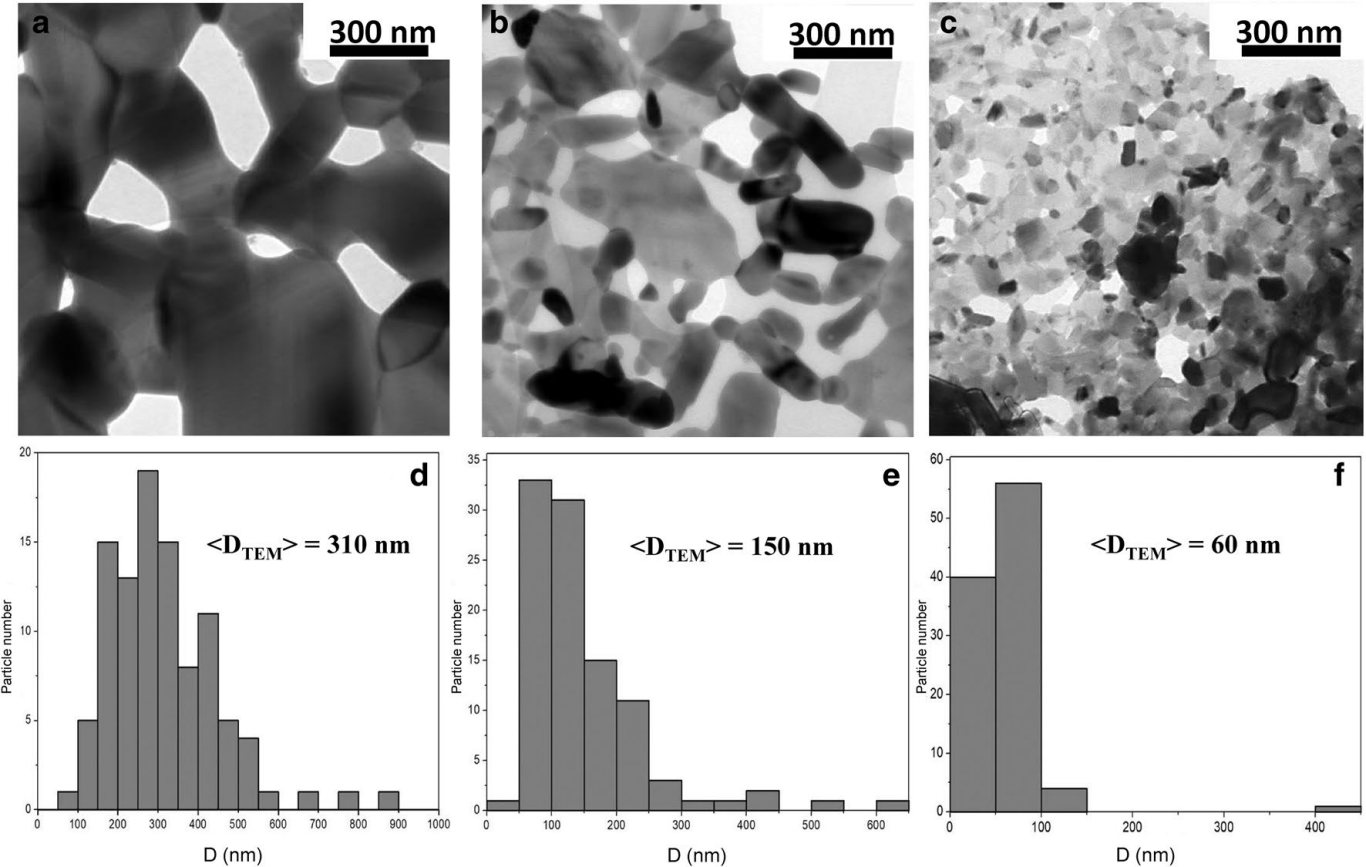
Bright field TEM images of (**a**) SFO_A_, (**b**) SFO_B_ and (**c**) SFO_C_ showing that they are composed of particles having a platelet shape. The corresponding size distributions are shown in the histograms of panels (**d**–**f**) respectively.

The Rietveld analysis on X-Rays powder diffraction (XRPD) data revealed that crystallite size along the c-axis can be modulated by suitable thermal treatments^6^. It specifically showed that the observed peak broadening in our samples is likely to originate from an anisotropic crystallite shape. Furthermore, the agreement between the average diameter from Rietveld analysis and Williamson-Hall plot (< *D*_*XRD*_ > and < *D*_*XRD*_^*WH*^ > in Table S1) suggests almost strain-free crystallites, with concomitant absence of structural distortions. The evolution of crystallite size distributions along different orientations focused our attention on the resulting particle size, whose analysis is not straightforward as the samples obtained by sol–gel self-combustion consist of particles´ aggregates with a porous structure^6,9,21^. TEM was used for investigating in more detail the inner structure of tested samples, and typical bright field images are reported in Fig. 3a–c (for detailed description of the analysis see Supplementary Material Sect. 2). Samples are composed by particles having a platelet shape. Platelets are linked together to form porous aggregates having different sizes and shapes. In order to estimate the size distribution of the platelets, the area of 100 particles was manually measured for each sample using the *ImageJ* software^22^. A representative size was attributed to each measured platelet taking as value the diameter of an equivalent circle having the same particle area. Results for samples SFO_A_, SFO_B_ and SFO_C_ are summarized in Fig. 3d–f respectively, and the average diameter values are also reported. Increasing the treatment temperature, the size of the particles and the width of their distribution increase. From the comparison between *V*_*TEM*_ and *V*_*XRD*_, Table 1, results that in case of SFO_A_ and SFO_B_ samples, the average number of crystallites per particle indicates the polycrystalline nature of these platelets. On the contrary, SFO_C_ has a particles volume comparable with the average crystallites volume, suggesting that a large part of the platelets is single crystal. We point out that the apparent size from TEM and X-ray diffraction is not the same, as in the latter we are measuring coherent scattering blocks (which can be smaller than normal platelets): in addition, small misorientation of these scattering domains would be accounted for by diffraction.

**Table 1:** Particle (< *D*_*TEM*_ >) and crystallite (*< D*_*XRD*_* >*) average size (nm) obtained from TEM and XRPD analysis, respectively, together with the corresponding calculated volumes.

Sample	< D_TEM_ > (nm)	< D_XRD_ > (nm)	< D_TEM_ > / < D_XRD_ >	V_TEM_ (nm^3^)	V_XRD_ (nm^3^)	N_C_ (V_TEM_ / V_XRD_)
SFO_A_	310 (10)	132 (11)	$\cong 2.4$	15,598 $\times$ 10^3^	1204 $\times$ 10^3^	$\cong 13$
SFO_B_	150 (20)	88 (9)	$\cong 1.7$	1767 $\times$ 10^3^	356 $\times$ 10^3^	$\cong$ 5
SFO_C_	60 (20)	63 (6)	$\cong 1$	113 $\times$ 10^3^	130 $\times$ 10^3^	$\cong 1$

The ratio between the volumes stands for the average number of crystallites within a particle (*N*_*C*_). Uncertainties in the last digit are given in parenthesis.

### Crystallites’ size analysis by line profile method

#### Hexaferrites

**Figure 4:**
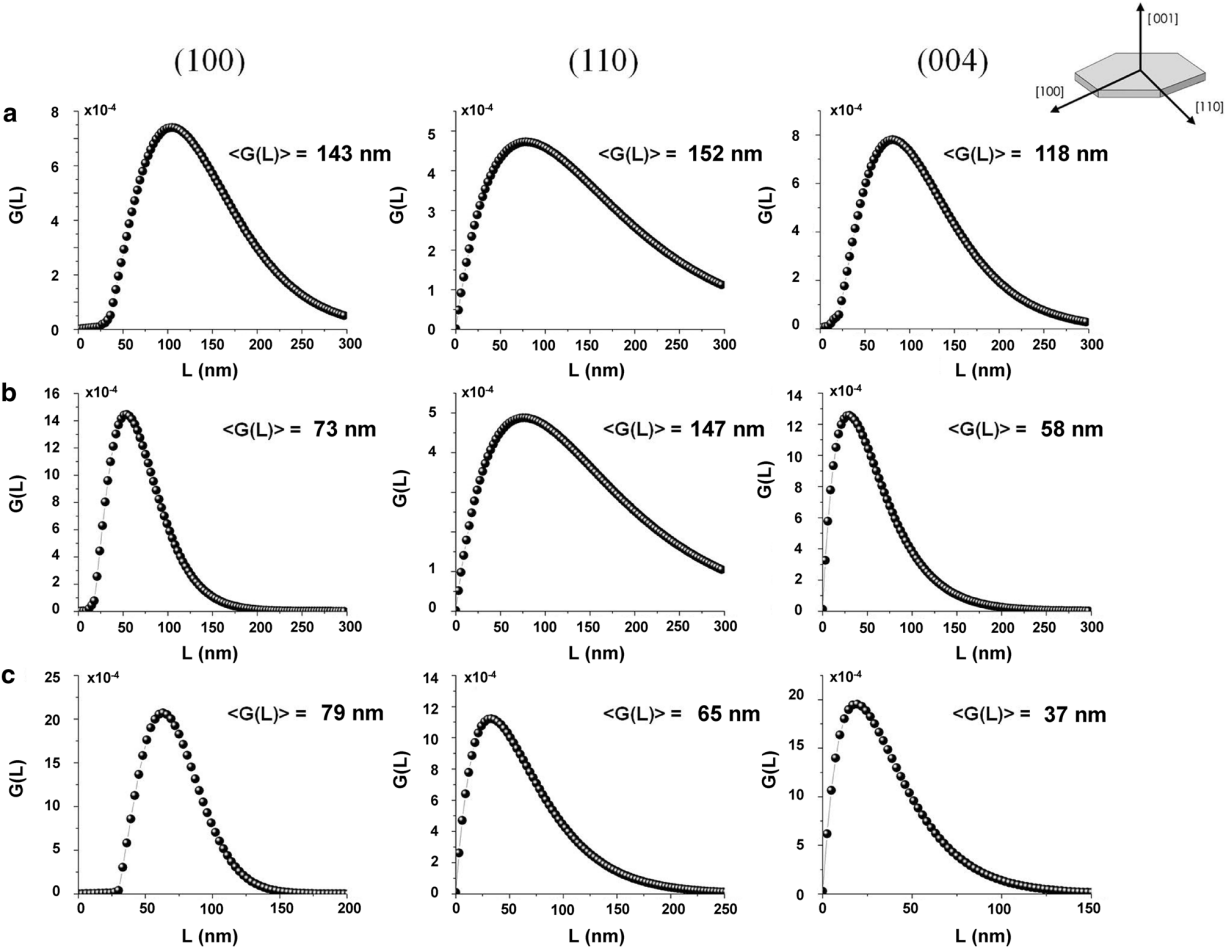
Volume-weighted crystallite size distribution *G(L)* for selected reflections (100), (110), (004), of (**a)** SFO_A_, (**b**) SFO_B_, (**c**) SFO_C_.

Crystallites in real powder samples form polydisperse systems, as we have also noticed from previous analysis. As X-rays methods are sensitive to blocks of coherent scattering, a thorough analysis of powder diffraction data is necessary to describe the fine nanostructure. Here, their sizes are discussed through the characterization by the volume-weighted crystallite size distribution function *G(L)*^23^, which may be interpreted as the probability density of finding a crystallite of an assumed shape and size, taken with a weight proportional to its volume, in an analyzed sample. Having prismatic crystallite shape, one can calculate the mean volume-weighted crystallite sizes (the mean edge length in the directions [100], [110] and [001]). Hereby we have selected all three samples of SFO with different granulometry, in form of anisotropic platelets (see Ref.^6^), in order to assess the validity of the procedure to get accurate crystallite-size distributions for nanoscale materials. To evaluate the anisotropic orientation of the ferrites´ crystallites, a line profile analysis of the XRPD data as performed, for selected peaks. The tested SFO samples do not contain convenient (pure) higher orders of diffraction from the same set of crystal planes, and therefore the separation of line-broadening contributions from size and distortion cannot be made. At the same time the observed broadening of the diffraction lines is more likely to be due to size effects by verifying the average crystallite shape from the analysis of several lines. The volume-weighted crystallite-size distribution functions *G(L)* along defined crystallographic directions are compared in Fig. 4. The typical form of a crystallite size distribution is the log-normal one. A characteristic feature of all obtained size distributions was their unimodality. In most cases, such a distribution can be attributed to some defined process of particle formation. The difference between the average calculated sizes and values extracted from Rietveld refinement for selected peaks is within acceptable limits (considering that the procedure of instrumental correction is different between these methods) and in agreement with the mean sizes obtained from the corresponding set of planes by Debye–Scherrer equation, as reported in Table 2. The trends of the volume averaged crystallite size for the two different modeling techniques are very similar, with a small offset in the absolute sizes. While a possible disagreement with Rietveld, as for instance in case of (110) reflection for SFO_B_ could be related to the correct determination of the background from both sides of selected reflections on the distance 1 degree of 2theta in each direction. Nevertheless, the excellent agreement between the two techniques confirms the relevance of the method. From the analysis of peak broadening, it is clear that there is a specific dependence of the size along [001] on the synthesis approach, resulting in the formation of platelet-like crystallites for sol–gel synthesized SFO^6,21^. This feature opens the way for the use of such a method in the design of nanocrystallites with preferential shape. As we know, the complex crystal structure of SFO (depicted in Fig. 1), is at the heart of the ferromagnetic behavior of SFO^12^, thus the design of optimized samples whose shape and size features can be tuned is critical for applications (e.g. permanent magnets related). We point out that crystallite size analysis is a powerful way to describe the shape anisotropy of crystallites, and further strengthens the results previously achieved.

**Table 2:** Crystallite sizes (nm) for selected reflections from: Rietveld refinement (*RR*) and line-profile analysis (*GL*) for SFO_A_, SFO_B_, SFO_C_; mean size calculated from Debye–Scherrer equation for each set of reflections ({100}, {110}, {001}) is also reported for comparison (<*DS*>).

Sample	RR /GL (nm)			< DS > (nm)		
	(100)	(110)	(004)	{100}	{110}	{001}
SFO_A_	146(7)/143(6)	152(8)/152(8)	134(3)/118(6)	113(5)	131(6)	111(5)
SFO_B_	91(5)/73(3)	118(6)/147(7)	68(5)/58(3)	84(4)	90(5)	61(3)
SFO_C_	74(4)/79(4)	69(4)/65(3)	52(2)/37(2)	61(3)	63(3)	49(2)

Uncertainties in the last digit are given in parenthesis.

#### Feasibility for hexaferrite/spinel nanocomposite-system

**Figure 5:**
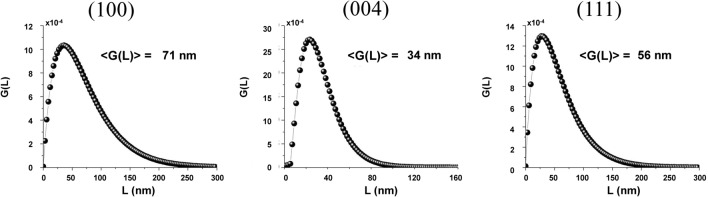
Volume-weighted crystallite size distribution *G(L)* for selected reflections of SFO ((100), (004)) and CFO (111) in the nanocomposite; for comparison corresponding Rietveld analysis values were respectively 70(7), 45(6) and 67(5) nm^6^.

To assess the validity of the procedure to get accurate crystallite-size distributions for nanoscale powder materials, and apply it to complex nanostructures, we have verified, as illustrated in Fig. 5, the accuracy of the method in case of a nanocomposite (with nominal composition SrFe_12_O_19_/CoFe_2_O_4_ 40/60 w/w %). These results are in perfect agreement with Rietveld analysis (see caption of Fig. 5 for a comparison), and a more plate-like morphology can be highlighted for SFO nanocrystallites as compared to the single-phase systems. These results are promising for the application of such line-profile analysis to more complex systems, where several different crystal phases can overlap, without losing the information of respective structures.

### Activation volume

**Figure 6:**
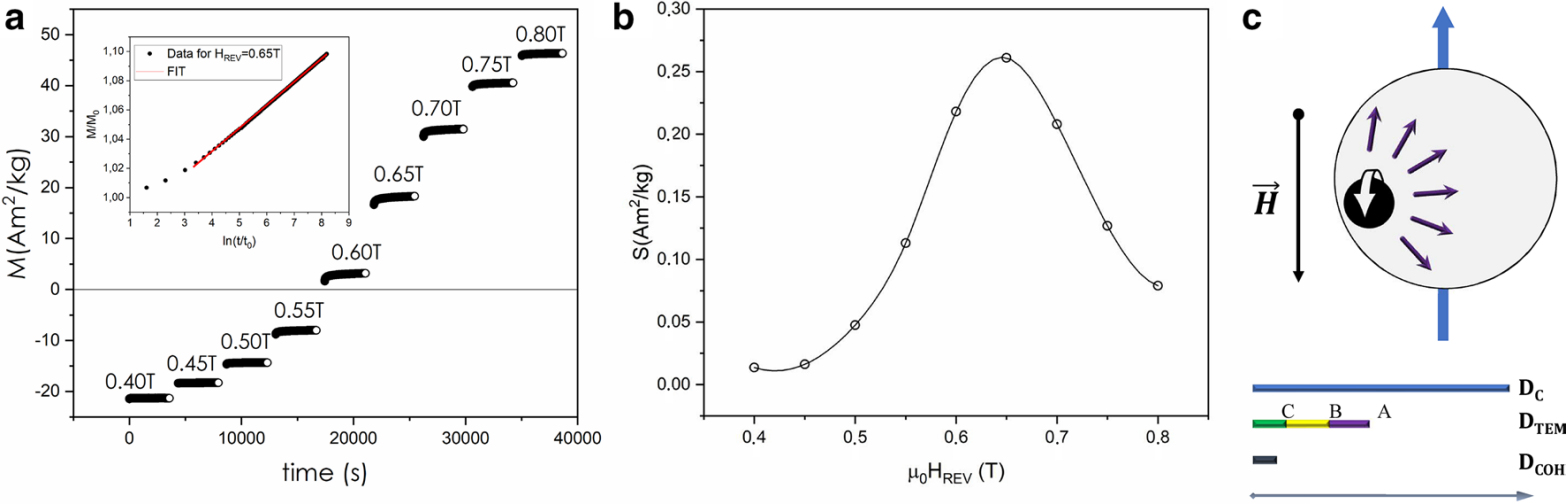
(**a**) Time dependent magnetization curves for SFO_C_ measured at different values of reverse field *H*_*REV*_ (indicated next to the experimental data) after saturation at − 5 T, at 300 K (Magnetization is normalized to the weight of sample); for clarity, the inset shows the experimental data (black circles) for 0.65 T field with the best fit (red line) (Magnetization is normalized to the initial value *M*_*0*_ = *M(t*_*0*_*)*); (**b**) corresponding magnetic viscosity (*S*) as a function of the reverse field for SFO_C_ (line is a guide to the eye); (**c**) scheme of activation mechanism with details of physical/magnetic length scales.

As seen in Fig. 2, determining the size of magnetic domains as well as the correct estimate of physical volumes is fundamental to describe such complex systems and obtain a clear understanding both of the magnetic interparticle interactions and the structural order. The magnetic behavior of SFOs samples was studied in detail recently, with particular focus on the reversal processes of the magnetization, with the aim of investigating the irreversible component of the susceptibility (*χ*_*irr*_) (Fig. S3 as an example for SFO_C_)^6^. To gain a deeper understanding on the magnetization reversal mechanism in this type of ferrites-based nanosystems, we performed magnetic relaxation measurements in a reverse field (*H*_*REV*_ ) after saturation in a given direction^17^. The so-called magnetic viscosity (*S*) was estimated for each *H*_*REV*_ considering $M\left( t \right) \propto Sln\left( t \right)$ (see Fig. 6 and Supplementary Material for further details) and in turn the activation volume (*V*_*ACT*_) was obtained. Since it can be defined as the smallest volume of material that reverses coherently in an event^18^, this parameter denotes the ´magnetic´ volume involved in the reversal process. Our values of *V*_*ACT*_ (see Table S3) correspond to a sphere with a diameter of ~ 30 nm, defined as coherent diameter (*D*_*COH*_), which describes the upper limit for a system´s magnetization to reverse by coherent rotation. These values are rather constant and small in spite of the huge difference in physical volume by particles (almost 10 times bigger for SFO_A_ than SFO_C_), suggesting that the magnetization reversal mechanism remains unchanged for all the systems (in agreement with our claim to be in the single-domain regime)^24^. Eventually, *V*_*ACT*_ turns out to be much smaller than the physical volume from XRPD and TEM analysis (*V*_*XRD*_ and *V*_*TEM*_ in Table S3). Therefore, we can conclude that the switching process does not occur only by coherent rotation. Note that results achieved by using different magnetometers (Fig. S4) gave a rather similar value of *D*_*COH*_. In this regard, it is extremely important to define the critical diameter for a single-domain particle (*D*_*C*_), in order to identify the most plausible reversal process. From our analysis (see Supplementary Material), we could deduce that the obtained *V*_*ACT*_ involves an incoherent rotation mechanism, since the D_C_ (~ 0.8 µm) is far from that of our particles, that is the formation of domain walls is no longer energetically favored, and a single domain configuration is obtained. Such results may be explained by the formation of interaction domains^25,26^. We assume that individual crystallites take part into one interaction domain, which extend over the interconnected particles due to the heterogeneous microstructure of these materials^27,28^. While X-rays methods are only sensitive to fine microstructure of domains (crystallites), the magnetic relaxation measurements gave evidence of complex phenomena which may occur in nanostructured SFO. As a result, by optimizing the nanometric size of SFO grains, one could prevent the switching to multidomain reversal processes and hence preserve high coercivity in those materials.

Generally speaking, magnetization reversal may occur through a series of local processes, such as nucleation, propagation and pinning-depinning of the domain walls^29^. In case of single-domain ferrite particles, the activation mechanism is nucleation-mediated, and initiated by the change of magnetization of a smaller volume than the overall one that reverses magnetically (as depicted in Fig. 6c)^29^.

The gap between the critical magnetic and physical diameter hints at incoherent modes as concomitant events for the reversal of magnetic domains, probably owed to inhomogeneities in the material and asperities at the surface, that becomes relevant when particle size increases^25^, resulting in a deviation from a uniformly magnetized state.

Therefore, we can conclude that the magnetization reversal process is quite complex in this kind of systems, while an effort to reduce the size within the nanoscale play a key role in the interplay between microstructure and magnetic properties in ferrites.

## Conclusion

Understanding the complex relation between structure, morphology and magnetic properties, is fundamental to design and develop future applications. Line-profile analysis of selected XRPD patterns of SrFe_12_O_19_ confirms the anisotropic shape of nanocrystallites obtained by our synthesis method. In combination with TEM analysis, the polycrystalline nature of such particles was evidenced, subsequently confirming that size of SFOs explored in this work are below the critical single-domain diameter, despite evidence of crystallites growth. On this basis, we propose an irreversible process for the magnetization based on the formation of interaction domains constituted by the interconnected crystallites. Our results demonstrate the intimate correlations between particle morphology, crystal structure, and crystallite size existing at the nanoscale. This study intends to clarify the reversal magnetization process of hard nanostructured magnetic materials, and determine the role played by microstructural features in the resulting magnetic behavior.

## Methods

### Sample preparation

Samples were synthesized following a sol–gel self-combustion approach via citric acid as chelating agent/fuel, reported in Ref.^6^. The synthesis conditions were optimized to obtain three samples with different sizes (SFO_A_, SFO_B_, SFO_C_), obtained by a proper annealing treatment at different temperatures temperature (respectively from 1000, 900, 800 °C). Table S1 sums up the magnetic properties, which were found to be relatively similar. A nanocomposite SrFe_12_O_19_/CoFe_2_O_4_ 40/60 w/w % was also prepared in a similar way.

### Structural characterization

Diffraction patterns were measured on a Bruker D8 powder diffractometer using a CuKα radiation (λ = 1.5418 Å) and setting the detector slit width to 0.2 mm. Data were collected in the 2θ range 10–140° by using a VANTEC counter. The temperature during the recording of data was maintained at 23 ± 1 °C. The reflections were measured by the step-scanning technique, with steps of 0.013° (2theta) for all tested samples; measurements were made up to − 2.5 and + 2.5° (2theta) from maximum peak. For each peak, a total of 106 quanta were counted, and for the tails around 3000 quanta. Several experimental peaks (separated or partially overlapping) were selected for further simultaneous analysis: (100), (110) and (004), occurring at Bragg angles close to those of the registered lines of SFO. Experimental intensities were corrected for the Lorentz-polarization factor and the background removed with a linear variation assumed. Corrections for instrumental and spectral broadening were made with NIST standard LaB_6_ (NIST 660b). The pure diffraction lines were obtained using the LWL (Louer-Weigel-Louboutin) deconvolution method^30,31^. The method was implemented in the profile analysis program *PROFIT-software*^32^. From a fit of the measured intensity data for sample and standard with a pseudo-Voigt function, the corresponding correct line-profile f(x) was extracted. The size distribution functions *G(L)* were determined from f(x) by following a procedure proposed in Ref.^23^. For more details see Supplementary Material. Complementary to the line profile analysis, Rietveld analysis was performed on XRPD data by using the *FULLPROF* program^33^ (the details can be found in Maltoni et al.^6^). Briefly, in the Rietveld model, the diffraction peaks were described by a modified Thompson–Cox–Hastings pseudo-Voigt function^34^. A LeBail refinement of data was performed on a NIST LaB_6_ 660b standard to account for the instrumental contribution to the peak broadening. From the calculated FWHM (full width at half the maximum intensity of the peak), the volume-weighted average size of coherently scattering crystalline domains can be calculated using the Debye–Scherrer equation:

$$< D_{XRD} > = \frac{K \cdot \lambda }{{FWHM \cdot \cos \theta }}$$

where λ is the X-ray radiation wavelength, K is the shape factor (0.8–1.2, typically equal 0.9), θ is the Bragg angle. This was applied for: selected reflections, the corresponding set of planes and the whole pattern (10–90°).

For comparison, average crystallite size was also calculated by Williamson-Hall plot^35^:

$$< D_{XRD} > = \frac{K \cdot \lambda }{{\left( {FWHM - 4\varepsilon \tan \theta } \right) \cdot \cos \theta }}$$

where ε is the microstrain.

Additionally, TEM analysis was carried out using a Philips CM200 microscope operating at 200 kV and equipped with a LaB_6_ filament to obtain information about particles´ morphology and size distribution.

### Magnetic characterization

Magnetization relaxation measurements were performed by two different instruments: a Physical Property Measurement System (PPMS)-Vibrating Sample Magnetometer (VSM) from Quantum Design, equipped with a superconducting magnet of 9 T, and a MicroSense Model 10 VSM equipped with an electromagnet generating a maximum field of 2 T the sample was saturated in a field (µ_0_H_MAX_: − 5 T and 2 T, respectively for each instrument), then a reverse field (*H*_*REV*_) was applied to bring the sample into the switching region (around *H*_*C*_ of the sample) and then the magnetization decay was recorded as a function of time for 60 min. The measurements were carried out at 300 K. The corresponding activation volumes were evaluated from those measurements as described in Supplementary Material.

## Supplementary Information

Supplementary Information.
