# Supplementary material for: Complex correlations between microstructure and magnetic behavior in SrFe12O19 hexaferrite nanoparticles
Source: Sci Rep. 2021 Dec 2;11:23307. doi: 10.1038/s41598-021-02782-2 (PMC8639738; doi:10.1038/s41598-021-02782-2)
Supplement: Supplementary file 1 — Supplementary Information. [file 41598_2021_2782_MOESM1_ESM.pdf]

Supplementary Material

Complex Correlations between Microstructure and Magnetic Behavior in $SrFe_{12}O_{19}$ Hexaferrite Nanoparticles

P. Maltoni[1*], S. A. Ivanov[1,2], G. Barucca[3], G. Varvaro[4], D. Peddis[4,5], R. Mathieu[1*]

1 Department of Materials Science and Engineering, Uppsala University, Box 35, SE-751 03 Uppsala, Sweden

2 Department of Chemistry, M.V. Lomonosov Moscow State University, Leninskie Gory 1/3, Moscow 119991, Russia

3 Department SIMAU, Universita Politecnica delle Marche, Via Brecce Bianche12, 60131 Ancona, Italy

4 Istituto di Struttura della Materia-CNR, nM2-Lab, 00015 Monterotondo Scalo (RM), Italy

5 Department of Chemistry and Industrial Chemistry, nM2-Lab, Universita degli Studi di Genova, Via Dodecaneso 31, 1-16146 Genova, Italy

Table of Contents

1.Sample Details 1

2.TEM Analysis 2

3.Line Profile Analysis 3

4.Relaxation Measurements 4

References 7

1.Sample Details

Table S1. Average size from XRD Rietveld analysis and Williamson-Hall plot (<D_{XRD}> and <D_{XRD}^{WH}>), saturation magnetizations (M_S), reduced remanence magnetization M_R/M_S and coercive field (H_C).

Sample	<D_{XRD}> (nm)	<D_{XRD}^{WH}> (nm)	M_S (Am^2/kg)	M_R/M_S	H (kA/m)
SFO_A	132(11)	134(12)	67.3(5)	0.49	469(3)
SFO_B	88(9)	88(8)	64.9(3)	0.50	480(2)
SFO_C	63(6)	64(6)	63.6(7)	0.50	468(2)

2.TEM Analysis

The acquired TEM images were analyzed in order to obtain a statistically relevant description of particles´ size. The investigated samples consist of platelets linked together to form porous aggregates, which makes the evaluation challenging. For these reasons, the area of the platelets was estimated by manually tracing the perimeter of a particle: Fig.S1 illustrates the identification of the grains´ boundaries; we point out that platelets are identified as different if separated by an evident grain boundary and only regions where platelets were well detectable (not severely agglomerated/superimposed) have been analyzed. A representative size was attributed to each measured platelet taking as value the diameter of an equivalent circle having the same particle area. The average diameter values (<D_{TEM}> (nm)), which were taken as representative size of the platelets, are obtained as arithmetic averages on 100 platelets. The associated uncertainty is estimated by considering the uncertainty in measuring the area, i.e. in manually drawing the correct perimeter of the platelets.

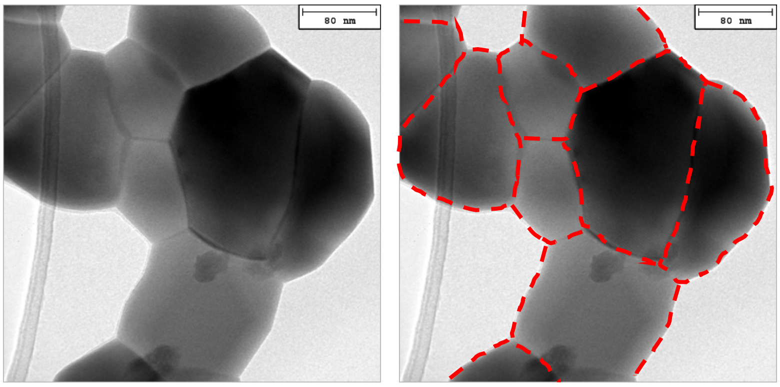

Fig. S1 Bright field TEM images of SFO_A showing the boundaries´ identification.

In order to analyze further the size distribution of the platelets we used the fitting options of the OriginPro 8.5 software [*OriginLab Corporation. One Roundhouse Plaza, Northampton, MA 01060. 1-800-969-7720. www. OriginLab.com.*].

The data set for all the samples resulted in a size-distribution typical of a Log-normal distribution:

$$y = y_0 + \frac{A}{w\,x\sqrt{2\pi}} \bullet e^{-\frac{\left(\ln \frac{x}{x_c}\right)^2}{2w^2}} \quad (1)$$

where A is a pre-factor, x is the platelets diameters, x_c is the median of the diameter and w is the standard deviation of the natural logarithm of the variable x. The fitting curves and the parameters obtained by the convergence procedure for the three samples are shown in Fig.

S2. Considering these size distributions, it is possible to calculate the average value of the diameter using the following equation:

$$< x > = x_c \, e^{\frac{w^2}{2}} \quad (2)$$

The obtained values are comparable with the arithmetic averaged diameter values $<D_{TEM}>$ previously obtained, as shown in Table S2.

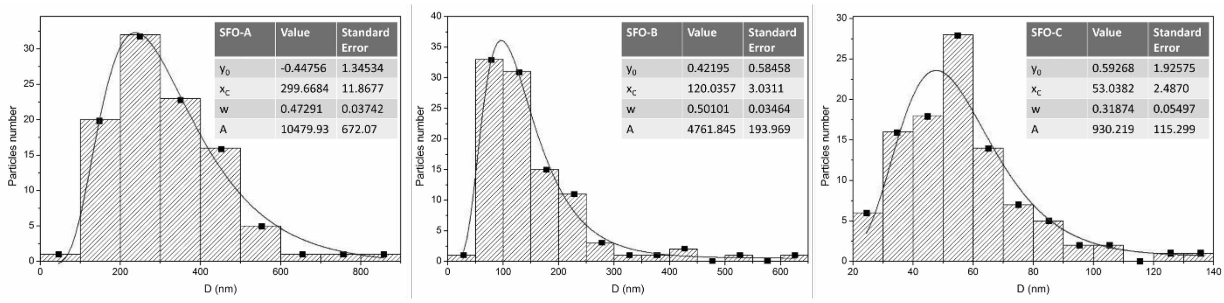

Fig. S2 TEM analysis of Log-normal size-distribution.

Table S2. Comparison between ($<D_{TEM}>$) and ($<x>$) values. (Uncertainties in the last digit are given in parenthesis)

Sample	$<D_{TEM}>$ (nm)	$<x>$ (nm)
SFO_A	310(10)	340(20)
SFO_B	150(20)	140(10)
SFO_C	60(20)	58(20)

3.Line Profile Analysis

Calculation of G(L) distributions was performed using a deconvolution approach (LWL) by a linear programming method [1–3]. This study was performed on a selected set of reflections (100), (110) and (004) to determine the size of hexagonal crystallites, by modelling individual line profiles.

The true line profile *f(x)*, representative of only sample microstructure, could be obtained by the deconvolution of the following equation:

$$h(x) = \frac{1}{2\pi} \int_{-\infty}^{+\infty} f(y) g(x - y) dy \qquad (1)$$

where *h(x)* is the experimental line profile, and *g(x)* the instrumental one, representing the geometric aberrations. The line profiles were approximated by processing the powder

diffraction data with PROFIT computer program [4]. According to Fourier analysis, *f(x)* can be expressed as:

$$f(x) = a_0 + \sum_{n=1}^{\infty} \left(a_n \cos 2\pi n x\right) \qquad (2)$$

where *x* is the reciprocal-space variable, *n* the harmonic number and a_n the coefficients. The Fourier cosine series were then obtained by deconvoluting *g(x)* from *h(x)* by the LWL method, knowing that they relate to the analytical function used to model experimental line profiles.

This approach is based on the existence of a hypothetical column of meshes of length L (known as Fourier length) normal to the diffracting planes hkl:

$$L = \frac{n\lambda}{2(\sin\theta_2 - \sin\theta_1)} \qquad (3)$$

where *n* is the harmonic number of the Fourier coefficients, λ the wavelength, θ_1 and θ_2 the extreme angles of the angular interval defining the line.

The coefficients can be reformulated into a distribution *P(L)*, since they are proportional to the lengths of columns of unit cells in the direction of the diffraction vector, whose mean $\bar{M}$ corresponds to the area-weighted mean apparent size. The corresponding volume-weighted distribution (known as crystallite-size distribution function) is here defined:

$$G(L) = \frac{L \bullet P(L)}{\bar{M}} \qquad (4),$$

The corresponding volume-weighted mean corresponds to:

$$< G(L) > \; = \varepsilon_\beta \quad (5)$$

which is the average apparent size perpendicularly to the considered hkl planes (linked to the integral-breadth apparent size). This distribution function mean gives an estimate of size.

Note that a least-squares procedure, with a stabilization scheme, was used for smoothing of crystallite-size distributions. No particular analytical expression is assumed for real X-ray diffraction reflections.

4.Relaxation Measurements

The time relaxation of the magnetization stems from the thermally activated reversal of the magnetization against the energy barriers of the system [5]. It can be described by the logarithmic (or quasi-logarithmic) decay of the magnetization in function of time (*M* versus *t*) according to:

$$M(t) = \; Mo \pm S\, ln\, (\frac{t}{t_0}) \qquad (1)$$

where S is the so-called magnetic viscosity coefficient, t_o is the reference time, M_o is a constant ($M(t=0)$), and the $\pm$ sign describes whether M is increasing or decreasing with time. The magnetic viscosity S was measured in the range of $\mu_0 H_{REV}$ between 0.40 T and 0.80 T, around coercivity value (≈ 0.60 T) of the investigated samples. The time dependence of magnetization for SFO_C is shown in Fig.6a as an example. By fitting the data with equation (1), S was estimated at different values of the reverse field (Fig.6b): S is field-dependent ($S(H_{REV})$) and tends to reach its maximum near the coercive field, thus strengthening the interconnection between magnetic viscosity and $\chi_{irr}(H_{REV})$ (which can be seen as the energy barrier distribution). χ_{irr} (H_{REV}), shown for SFO_C in Fig.S3, was estimated from the derivative of $M(H_{REV})$ curve, see e.g. Ref.[6]

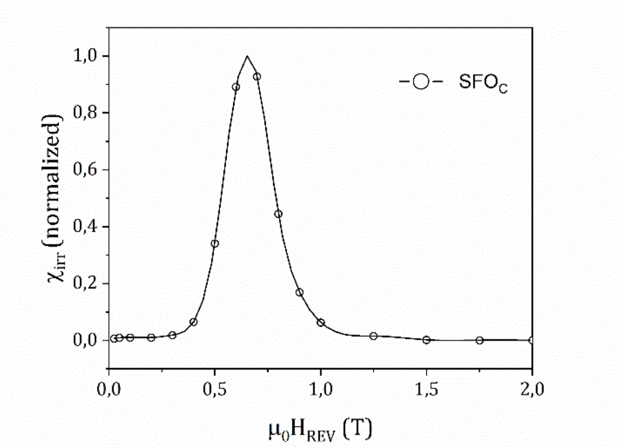

Fig.S3 Normalized switching field distribution (SFD) of SFO_C at 300 K (line is a guide to the eye).

Then, by combining the maximum value of magnetic viscosity with the irreversible susceptibility (χ_{irr}) (at the same field, from switching field distribution curve), an estimation of fluctuation field (H_F) could be obtained:

$$H_F = \frac{S}{\chi_{irr}} \quad (2).$$

This is related to the activation volume (V_{ACT}), which can be defined as the smallest volume of material that reverses coherently in an event, and for a single-domain particle with uniaxial anisotropy can be expressed as [7]:

$$V_{ACT} = \frac{k_B T}{M_S H_F} \quad (3)$$

Where k_B is the Boltzmann constant, T is the temperature, and M_S the saturation magnetization. This parameter denotes the basic ´magnetic´ volume involved in the reversal process. V_{ACT} was calculated for each sample, and its values are reported in Table S3. Additionally, data of SFO_B sample were evaluated with the aid of two protocols performed by a PPMS and VSM magnetometers (Fig.S4a and b), respectively. We point out that the agreement between the data lies within the experimental uncertainty given by the respective techniques.

The found values are in good agreement with ones obtained experimentally [8] and by micromagnetic models for this kind of hard ferromagnetic materials (radii between ~10-30 nm) [9,10], according to the following equation for a spherical particle:

$$\frac{D_{COH}}{2} = 5.099 L_{ex} \quad (4)$$

where $L_{ex} = \sqrt{\frac{A}{K_{eff}}}$ is the so called exchange length, and A and K are the exchange stiffness and the effective anisotropy constant, respectively [11]. However, microstructural and surface effects on particle magnetic anisotropy are neglected here. Additionally, it is noteworthy that the viscosity in a system of particles depends on a number of parameters, such as the reversal mechanism, the interparticle interactions, the particle volume, the anisotropy field, and the easy axis distribution that affects the final evaluation of coherent size, as for instance local effects tend to reduce the experimental V_{ACT} [12]. In this regard, it is extremely important to define the critical size for a single-domain particle (D_C), in order to identify the most plausible reversal process. According to [13]:

$$D_C = \frac{1}{N} 24 \frac{\sqrt{A K_{eff}}}{\mu_0 M_S^2} \quad (5)$$

where N is the shape demagnetization factor, by assuming N = 1/3 (typical of spherical systems) and other parameters typical of M-type ferrite [10], the critical diameter is found to be ~0.8 μm, in agreement with the reported experimental values (between 0.5 and 1.5 μm) [14].

Table S3. Activation volume (V_{ACT}) extracted by relaxation measurements by PPMS and corresponding diameter ($\langle D_{COH} \rangle$) for $SFO_{A,B,C}$ at 300K. In blue the results obtained by VSM are also shown. The average size obtained from XRD Rietveld and TEM analysis together with the corresponding volumes are provided for comparison ($\langle D_{XRD} \rangle$, $\langle D_{TEM} \rangle$ and V_{XRD}, V_{TEM}, respectively). (Uncertainties in the last digit are given in parenthesis)

	Physical Size				Magnetic Size	
**Sample**	**$\langle D_{XRD} \rangle$ (nm)**	**V_{XRD} (nm^3)**	**$\langle D_{TEM} \rangle$ (nm)**	**V_{TEM} (nm^3)**	**V_{ACT} (nm^3)**	**$\langle D_{COH} \rangle$ (nm)**
SFO_A	132(11)	1204×10^3	310(10)	15598×10^3	13×10^3	30(6)
SFO_B	88(9)	356×10^3	150(20)	1767×10^3	11×10^3 14.5 ×10^3	28(6) 30(6)
SFO_C	63(6)	130×10^3	60(20)	113×10^3	8×10^3	25(4)

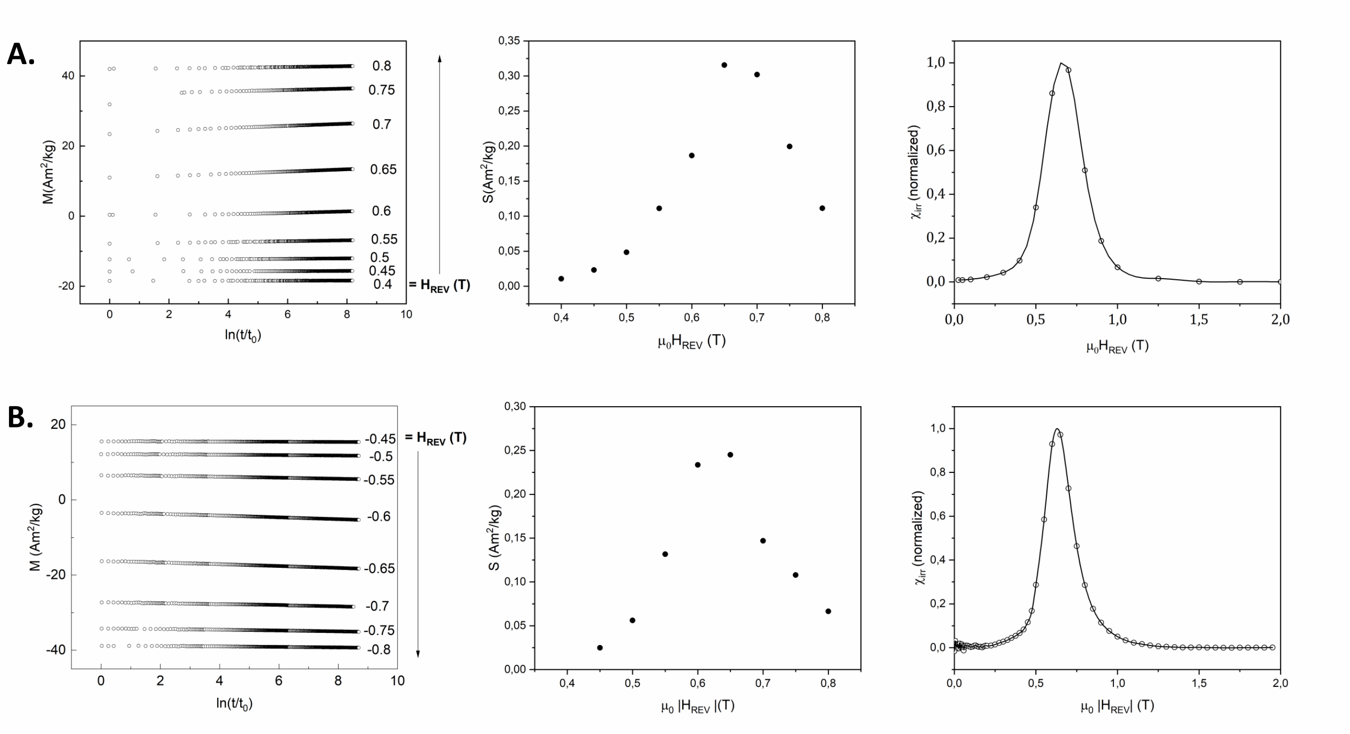

Fig.S4 Comparison between magnetic data of SFO_B acquired by PPMS (a) and VSM (b) magnetometers respectively, at 300K (lines are a guide to the eye).

References

[1] A. Le Bail, D. Louër, Smoothing and validity of crystallite-size distributions from X-ray line-profile analysis, J. Appl. Crystallogr. 11 (1978) 50–55. https://doi.org/10.1107/S0021889878012662.

[2] A. Le Bail, Defect and Microstructure Analysis by Diffraction, Oxford Uni, 1999.

[3] A. Le Bail, D. Louer, ETUDE PAR DIFFRACTION DES RAYONS X DE LA TRANSFORMATION D'UN HYDROXYNITRATE DE NICKEL NON STOECHIOMETRIQUE EN HYDROXYDE, Rev. Chim. Miner. 17 (1980) 522–532. http://pascal-francis.inist.fr/vibad/index.php?action=getRecordDetail&idt=PASCAL8130361045.

[4] S.A. Zhurov, V. V., & Ivanov, PROFIT computer program for processing powder diffraction data on an IBM PC with a graphic user interface., Crystallogr. Reports. 42 (1997) 202–206. http://dx.doi.org/10.1134/1.170588.

[5] S. Laureti, G. Varvaro, A.M. Testa, D. Fiorani, E. Agostinelli, G. Piccaluga, A. Musinu, A. Ardu, D. Peddis, Magnetic interactions in silica coated nanoporous assemblies of $CoFe_2O_4$ nanoparticles with cubic magnetic anisotropy, Nanotechnology. 21 (2010) 315701. https://doi.org/10.1088/0957-4484/21/31/315701.

[6] P. Maltoni, T. Sarkar, G. Varvaro, G. Barucca, S.A. Ivanov, D. Peddis, R. Mathieu, Towards bi-magnetic nanocomposites as permanent magnets through the optimization of the synthesis and magnetic properties of $SrFe_{12}O_{19}$ nanocrystallites, J. Phys. D. Appl. Phys. 54 (2021) 124004. https://doi.org/10.1088/1361-6463/abd20d.

[7] K. O'Grady, H. Laidler, The limits to magnetic recording — media considerations, J. Magn. Magn. Mater. 200 (1999) 616–633. https://doi.org/10.1016/S0304-8853(99)00499-0.

[8] K. Yamanaka, Y. Uesaka, T. Okuwaki, Magnetic viscosity of oriented barium ferrite media, J. Magn. Magn. Mater. 127 (1993) 233–240. https://doi.org/10.1016/0304-8853(93)90221-M.

[9] D. Sellmyer, R. Skomski, eds., Advanced Magnetic Nanostructures, Springer US, Boston, MA, 2006. https://doi.org/10.1007/b101199.

[10] J.M.D. Coey, Magnetism and Magnetic Materials, Cambridge University Press, 2001. https://doi.org/10.1017/CBO9780511845000.

[11] G. Herzer, Grain size dependence of coercivity and permeability in nanocrystalline ferromagnets, IEEE Trans. Magn. 26 (1990) 1397–1402. https://doi.org/10.1109/20.104389.

[12] V. Patel, M. El-Hilo, K. O'Grady, R.W. Chantrell, Nucleation fields in an exchange spring hard magnet, J. Phys. D. Appl. Phys. 26 (1993) 1453–1458. https://doi.org/10.1088/0022-3727/26/9/018.

[13] C. Kittel, Physical Theory of Ferromagnetic Domains, Rev. Mod. Phys. 21 (1949) 541–583. https://doi.org/10.1103/RevModPhys.21.541.

[14] F.H. Gjørup, M. Saura-Múzquiz, J.V. Ahlburg, H.L. Andersen, M. Christensen, Coercivity enhancement of strontium hexaferrite nano-crystallites through morphology controlled annealing, Materialia. 4 (2018) 203–210. https://doi.org/10.1016/j.mtla.2018.09.017.
